# TNF-Alpha Levels in Tears: A Novel Biomarker to Assess the Degree of Diabetic Retinopathy

**DOI:** 10.1155/2013/629529

**Published:** 2013-10-23

**Authors:** C. Costagliola, V. Romano, M. De Tollis, F. Aceto, R. dell'Omo, M. R. Romano, C. Pedicino, F. Semeraro

**Affiliations:** ^1^Department of Medicine and Health Sciences, University of Molise, Campobasso, Italy; ^2^Second University of Naples, Via Pansini 5, 80100 Naples, Italy; ^3^“Casa di Cura Villa Maria”, Campobasso, Italy; ^4^Department of Medical and Surgical Specialities, Radiological Specialities and Public Health, Eye Clinic, University of Brescia, Brescia, Italy

## Abstract

We assess the level of tumour necrosis factor alpha (TNF-alpha) in tear fluids and other serum parameters associated with diabetes in different degrees of diabetic retinopathy. We have performed a prospective, nonrandomized, observational study. Study population consisted of 16 healthy subjects (controls) and 32 type 2 diabetic patients: 16 affected by proliferative diabetic retinopathy (PDR) and 16 with nonproliferative retinopathy (NDPR, background/preproliferative). Body mass index, urinary albumin, blood glucose, HbA1c, and tear levels of TNF-alpha were measured in all subjects. The value of glycaemia, microalbuminurea, and Body mass index in diabetic retinopathy groups were higher than those in control group (*P* < 0.05). Glycemia in NPDR: 6.6 mmol/L (range: 5.8–6.3); in PDR: 6.7 mmol/L (range: 6.1–7.2); in control: 5.7 mmol/L (range: 4.9–6.1); microalbuminurea in NPDR: 10.6 mg/L (range: 5.6–20); in PDR: 25.2 mg/L (range: 17–40); in control: 5.3 mg/L (range: 2.6–10); Body mass index in NPDR: 26 Kg/m^2^ (range: 20.3–40); in PDR: 28 Kg/m^2^ (range 20.3–52); in control: 21 Kg/m^2^ (range 19–26). The TNF-alpha concentrations in tears increase with the severity of pathology and were lower in control group than in diabetic subjects. In the end, the level of TNF-alpha is highly correlated with severity of diabetic retinopathy and with nephropathy. Tear fluid collection may be a useful noninvasive method for the detection of proliferative diabetic retinopathy.

## 1. Introduction

Diabetes is a pathologic condition strongly associated with both microvascular (involving small vessels, such as capillaries) and macrovascular (involving large vessels, such as arteries and veins) complications. Microvascular complications include retinopathy, nephropathy, and neuropathy; whereas macrovascular complications include cardiovascular diseases, strokes, and insufficiency in blood flow to legs. These complications are triggered by the same culprit: elevated blood glucose levels. In fact, chronic hyperglycaemia plays a major role in the beginning of diabetic vascular complications through many metabolic and structural derangements, that is, the production of advanced glycation end products, abnormal activation of signaling cascades, elevated production of reactive oxygen species, and abnormal stimulation of hemodynamic regulation systems. Thus, metabolic control in diabetes can delay the onset and progression of these complications [1, 2].

Diabetic retinopathy (DR) is a leading cause of blindness and visual disability. DR is a serious consequence of long-standing and poorly controlled diabetes and the most important cause of vision loss in the working-age population in developed countries [3]. One hundred and fifty million of people in the world are affected, and in 10–15 years the World Health Organization predicts that the number of affected people will be doubled [4]. DR is classified according to the presence or absence of abnormal new vessels as nonproliferative (background/preproliferative) retinopathy and proliferative retinopathy [5]. Different studies reported that the prevalence of any retinopathy in diabetic patients is more than 30% [6–8]. Chronic hyperglycaemia drew metabolic and haemodynamic derangements, such as impaired blood flow regulation, increased vascular permeability, capillary basement membrane thickening, microaneurysm formation, microvascular cell death, and eventually widespread nonperfusion and ischemia of the inner retina [9], leading to dysfunctional responses in a range of cell types including neurons, glial cells, and supporting microvasculature [10]. While the precise pathogenesis of DR remains incompletely understood, inflammation and related processes are now thought to contribute to neuronal, glial, and microvascular lesions [10–12]. However, the role played by inflammation in DR has not yet been completely clarified [13, 14]. 

 TNF-alpha is an inflammatory cytokine, strongly correlated with insulin resistance and chronic inflammation [15]. Recently, the molecular mechanisms of TNF-alpha function have been intensively investigated. Many studies demonstrated increased circulating levels of TNF-alpha both in animals [16, 17] and humans [18–24], as well as in the retina of diabetic rat [25]. Moreover, TNF-alpha also has a role in the development of insulin resistance; in fact, it affects insulin sensitivity by changing the phosphorylation of insulin receptor substrate-1 and interferes with the insulin signaling cascade, thereby leading to insulin resistance [26, 27]. TNF-alpha is a potent mediator of the leukostasis induced by VEGF, interleukin-1 alpha, and platelet-activating factor in the retinal vasculature [28], and it also mediates the cell death/apoptosis of retinal neurons and vascular endothelial cells in DR [29]. These studies suggest that the retinal leukostasis and apoptosis mediated by TNF-alpha contribute to blood retinal barrier (BRB) breakdown in DR. Previous studies have shown that the level of soluble TNF receptors increases in the serum and vitreous of patients with proliferative diabetic retinopathy [30, 31]. These findings provide a potential link between inflammation and insulin resistance, further confirmed by the evidence that TNF-alpha inhibition prevents the pathologic events related to the development of early DR, including BRB breakdown [16]. 

The present study was designed to verify the feasibility of using the tear TNF-alpha assay as a novel biomarker to assess the degree of eye involvement in type 2 diabetic patients with and without proliferative diabetic retinopathy.

## 2. Patients and Methods

### 2.1. Subjects and Methods

The study was conducted in accordance with the tenets of the Declaration of Helsinki and the Institutional Review Board approved the study protocol. All participating subjects gave their informed consent after a detailed description of the procedure used and of the aim work. Thirty-two diabetic patients and 16 healthy subjects were recruited for this study. Diagnosis of type 2 diabetes was carried out according to the guidelines of American Diabetes Association [32]. Diabetic retinopathy classification and grading was performed according to the ETDRS criteria [33]. The study was conducted at the Operative Unit of Ophthalmology, “Casa di Cura Villa Maria”, University of Molise, Campobasso, Italy. Diabetic patients were divided into 2 groups: 16 patients presented nonproliferative retinopathy (NPDR, background/preproliferative retinopathy, 9 : 7, male : female; 54 years: range 49–67), whereas the remaining 16 patients were diagnosed as affected by proliferative diabetic retinopathy (PDR, 10 : 6, male : female; 59 years: range 52–73). Before study inclusion, each patient underwent a baseline eye check including best corrected visual acuity (BCVA) measured at 2 m with a standard ETDRS chart, biomicroscopy and fundus examination, applanation tonometry, fluorescein angiography, and evaluation of foveal thickness by optical coherence tomography (Spectralis HRA + OCT, Heidelberg Engineering, Heidelberg, Germany). The control group consisted of 16 healthy, age-matched, randomly selected University of Molise employers.

 Body height and weight and waist circumference were measured to calculate the Body Mass Index (BMI). The following formula was used: weight in kilograms/height in meters squared (kg/m²). The BMI results are classified as: (i) underweight (BMI < 18.5); (ii) healthy weight (BMI between 18.5 and 24.9); (iii) overweight (BMI between 25 and 30); (iv) obese (BMI > 30); (v) morbidly obese (BMI > 40). Blood glucose, HbA1c (NycoCard HbA1c test and NycoCard Reader, Axis-Shield PoC AS), blood urea nitrogen (BUN), and protein urine (Micral-Test, Roche Diagnostics Germany) were performed to all subjects. The occurrence of nephropathy was assessed measuring the glomerular filtration rate (GFR), which was calculated according to the Cockcroft formula [34]. A value of 90 mL/min/1.73 m² or higher was considered normal.

 Nonstimulated tears were collected from each eye from the inferior tear meniscus between the 6 o'clock and lateral canthus positions using a standard clinical Schirmer's strip. Following collection, the strip was immediately transferred into a sterile labelled tube and frozen at −40°C until analysed. The wet portion of each Schirmer's strip was then cut into smaller pieces and soaked in 50 *μ*L/5 mm of phosphate-buffered saline (PBS) for 3 h to elute tear proteins. TNF-alpha was measured using a 96-well plate coated with antibody specific for human TNF-alpha, according to manufacturer's instructions (Human TNF-alpha ELISA Development Kit PEP-900-K25, Li StarFish S.r.l.Cernusco S/N, Milan, Italy).

 Data were recorded in an Excel 2007 spreadsheet and analyzed by SPSS statistical software. For analysis of statistical differences, we used the Wilcoxon signed-rank test to evaluate the intragroup significance between variables. Conversely, the Mann-Whitney *U*-test was used to compare the control and DR groups. A *P* value of  <0.05 was considered statistically significant.

## 3. Results

**Figure 1:**
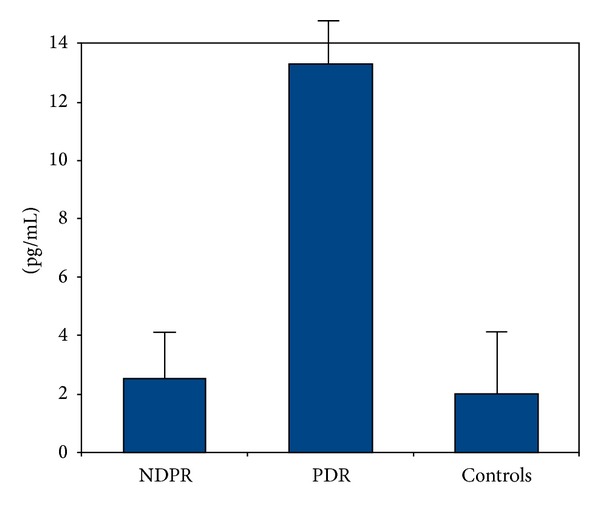
TNF-alpha levels in tears of the three different study groups. NPDR: non proliferative diabetic retinopathy; PDR: proliferative group diabetic retinopathy. The values are given as media and standard deviation.

 BMI, blood glucose, HbA1c, diabetes duration, BUN, protein urine, and GRF data are showed in Table 1. In diabetic patients, blood glucose levels were slightly higher than those recorded in the control group: NPDR: 6.6 mmol/L (range: 5.8–6.3); PDR: 6.7 mmol/L (range: 6.1–7.2); controls: 5.7 mmol/L (range: 4.9–6.1); *P* = not significant. The blood glucose difference between diabetic patients with and without proliferative retinopathy was also not significant. The HbA1c percentage was significantly higher in the groups of diabetics than in controls: NPDR: 7.2% (range: 6.5–11.5); PDR: 7.8% (range: 7.2–12.5); controls: 5.6% (range: 5.3–6.1); *P* < 0.05. However, there were no significant intragroup differences (NPDR versus PDR patients, *P* = not significant). Diabetic subjects exhibited levels of protein urine significantly higher (*P* < 0.001) than controls: NPDR: 10.6 mg/L (range: 5.6–20); PDR: 25.2 mg/L (range: 17–40); controls: 5.3 mg/L (range: 2.6–10). The intra-group difference (PDR versus NDPR) was also highly significant (*P* < 0.001). The values of BMI between diabetic and nondiabetic subjects were significantly different: NPDR: 26 Kg/m^2^ (range: 20.3–40); PDR: 28 Kg/m^2^ (range 20.3–52); control: 21 Kg/m^2^ (range 19–26); *P* < 0.05. Contrarily, the difference between diabetic patients with and without proliferative retinopathy was not significant. Duration of diabetes was higher in patients with PDR (*P* < 0.05). Lastly, diabetic patients showed a significant reduction (*P* < 0.001) of GFR: from 95 mL/min/1.73 m^2^ (73–91) of controls to 82 mL/min/1.73 m^2^ (46–89) and 64 mL/min/1.73 m^2^ (56–78) of NPDR and PDR patients, respectively. The intra-group difference (PDR versus NDPR) was also highly significant (*P* < 0.001). Tears TNF-alpha levels were lower in control than those in PDR group (*P* < 0.05) and the TNF-alpha concentration significantly increased along with the severity of pathology: control: 1.9 Kg/mL (range 1.1–6.9); NPDR: 2.8 pg/mL (range: 1.2–5.5); PDR: 13.5 pg/mL (range 9.2–21.7). (Figure 1).

## 4. Discussion

 To the best of our knowledge, this is the first report in which TNF-alpha in tears from patients with type 2 diabetes has been measured. The levels of TNF-alpha were associated with the degree of DR, being lower in NPDR and higher in PDR. These findings of ours tally with those reported by previous investigators which showed that proangiogenic cytokines are more highly represented than antiangiogenic cytokines in the tears of type 2 diabetic patients with retinopathy [35, 36]. 

 Several studies have shown the association between TNF-alpha and diabetic vascular damage [16, 37]. Kuo et al. demonstrated that the levels of soluble TNF 1 and 2 receptors are highly correlated with the severity of DR, suggesting a role of TNF-alpha in the development of DR [38]. Also other authors have found that the inflammatory marker TNF-alpha was associated with the severity of DR in type 1 diabetic participant with kidney disease [14]. Limb et al. demonstrated that the type 1 diabetic patients with PDR exhibited significantly higher serum levels of soluble TNF-receptors 1 and 2 than those recorded in patients without retinopathy or in healthy individuals. Our results strengthen the important role of TNF-alpha in DR. Furthermore, another important finding is the strong association between nephropathy and retinopathy; in fact, in our patients a significant correlation among HbA1c, body mass index (kg/m^2^), glomerular filtration rate (GFR), albuminuria, blood urea nitrogen, and levels of TNF-alpha in the tears has been found, further confirming that TNF-alpha tear levels may be considered a good predictor of microvascular complications. TNF-alpha is a central regulator of inflammation, and TNF-alpha antagonists may be effective in treating inflammatory disorders in which TNF-alpha plays an important pathogenetic role. Strong correlations between enhanced inflammatory biomarkers, including TNF-alpha, and the occurrence of diabetic retinopathy have been reported through extensive studies by many researchers [39, 40]. In the retina, it was shown that diabetes activates induction of proinflammatory mediators such as monocyte chemoattractant protein-1, interleukin-6, intercellular adhesion molecule-1, inducible nitric oxide synthase, matrix metalloproteinase-9, and TNF-alpha [40]. High glucose and advanced glycation end products stimulate monocytes/macrophages and their release of sTNF-alpha [41]. Serum concentrations of sTNF-alpha is increased in type 1/type 2 diabetic animals and diabetic patients with microangiopathy. Patients with proliferative DR show significantly higher serum TNF-alpha compared with that of nonproliferative DR patients [39]. TNF-alpha is elevated approximately 3-fold, and the receptors for TNF-alpha were increased by 40% in poor glycemic control retina compared with normal rat retina [42]. Altogether these findings indicate a role for TNF-alpha in DR pathogenesis and therapy and as a potential diagnostic target. 

 Early detection and treatment of diabetes can decrease the risk of developing its complications. Randomized trials have established the benefits of interventions to prevent or delay diabetes and reduce diabetes-related complications [43]. Screening for undiagnosed diabetes is generally considered to be safe. Most screening procedures begin with a risk assessment that relies on routinely collected demographic and clinical examination information followed by blood testing in high-risk individuals. There are also well-established and accepted diagnostic criteria for making a diagnosis of type 2 diabetes [44]. The American Diabetes Association has recommended that nondiabetic individuals ≥45 years of age be screened for diabetes at least every 3 years. Despite frequent screening and appropriate targeting of high-risk patients, followup of patients with abnormal results is uncommon and the yield of screening is low [45]. It is mandatory to diagnose DR as soon as possible, since early detection and timely treatment can prevent vision loss. Shortening the diagnostic delay, it is possible to act against the diabetes complications. It has been shown that an intensive treatment designed to keep glucose levels close to normal reduces the risk of developing long term complications, including retinopathy, and slow the progression of preexisting retinopathy in diabetic patients [46]. In this way we can reduce the number of diabetes patients with visual impairment [47] and the cost of diabetes related blindness that requires approximately $500 million/annually in USA [48]. For this reason there is a great interest to find biomarkers suitable for the prediction of vision loss as soon as possible [13, 14, 30, 31, 49].

 The proposed method does not replace eye exams by an ophthalmologist, but it could be seen as a “filter” to exclude diabetic patients without diabetic retinopathy. Furthermore, increasing rate of patients with diabetes soon will outpace the supply of eye care providers, and then some communities have poor or even no access to ophthalmologic care. It was seen that the tear levels of TNF-alpha increases in various inflammatory pathologies of the ocular surface (such as Sjogren's syndrome, rosacea, and dry eye) [50–52]. Once these diseases are ruled out, TNF-alpha can be used as a biomarker for DR screening. Thus, the medical costs could be reduced, and from a patient's perspective it could be favourable because the required pupil dilation may be uncomfortable. Last but not least, in this way all diabetic patients could be subjected to a very easy and noninvasive screening procedure to assess the presence of DR. In conclusion, data from this study indicate that TNF-alpha levels are associated with DR after adjusting for potential confounders. Level of TNF-alpha may be correlated with clinical disease severity and with predictors of kidney microvascular damage, BMI, and HbA1c. This finding needs to be replicated in prospective studies performed on a higher number of subjects.

## Figures and Tables

**Table 1:** Demographic and laboratory results of cohort.

	Controls Healthy subjects	Cases		*P* value
		Diabetic patient		
		NPDR	PDR	
No. of subjects	16	16	16	N.S.^#^ N.S.*
Age, yrs (range)	53 (47–58)	54 (49–67)	59 (52–73)	N.S.^#^ N.S.*
Gender (M/F)	(8/8)	(9/7)	(10/6)	N.S.^#^ N.S.*
BMI (Kg/m^2^)	21 (19–26)	26 (20.3–40)^#^	28 (20.3–52)^#^	<0.05^#^ N.S.*
Glucose (mmol/L)	5.7 (4.9–6.1)	6.6 (5.8–6.3)	6.7 (6.1–7.2)	N.S.^#^ N.S.*
HbA_1c_ (%)	5.6 (5.3–6.1)	7.2 (6.5–11.5)^#^	7.8 (7.2–12.5)^#^	<0.05^#^ N.S.*
DM duration (yrs)	Not available	14.8 ± 8.7^#^	16.5 ± 11.3^#∗^	<0.001^#^ <0.05*
BUN (mg/dL)	15 (6–20)	30 (24–48)^#^	44 (26–51)^#∗^	<0.001^#^ <0.001*
Protein urine (mg/L)	5.3 (2.6–10)	10.6 (5.6–20)^#^	25.2 (17–40)^#∗^	<0.001^#^ <0.001*
GFR (mL/min/1.73 m^2^)	95 (73–91)	82 (46–89)^#^	64 (56–78)^#∗^	<0.001^#^ <0.001*

BMI: Body Mass Index; DM: Diabetes Mellitus; BUN: Blood Urea Nitrogen; GRF: Glomerular Filtration Rate.

^#^Mann-Whitney *U*-test: diabetic patients versus controls; *Wilcoxon signed-rank test: patients with PDR versus patients with NPDR; NS: not significant.
